# Rapid Prototyping of a Micromotor with an Optical Rotary Encoder

**DOI:** 10.3390/mi8060174

**Published:** 2017-06-02

**Authors:** Da-Chen Pang, Yi-Wei Lai

**Affiliations:** Department of Mechanical Engineering, National Kaohsiung University of Applied Sciences, 415 Jian Gong Rd., Sanmin Dist., Kaohsiung 80778, Taiwan; bw016650@gmail.com

**Keywords:** micromotor, permanent-magnet synchronous motors, flexible print circuit, electroforming, optical encoder, optical fiber, rapid prototyping

## Abstract

This study proposed a rapid prototyping fabrication method for micromotors that allowed us to develop both 1 mm and 1.5 mm diameter permanent-magnet synchronous motors (PMSMs) with an optical rotary encoder. First, an integrated electroforming method was proposed for combining stator housing and flexible print circuit (FPC) coils to ease the manufacturing and assembly of micromotor components. This is particularly useful in the production of prototypes or small volumes of units. Second, an optical encoder was used to detect the rotational angle by means of a reflective code disk, an optical fiber, and a photo-detector. The micromotor was built with a code disk and an optical fiber. The code disk was designed to match the optical fiber and was made by photolithography and sputtering. Both the 1 mm and 1.5 mm diameter motors successfully achieved a rotational speed over 20,000 RPM and due to a 50 µm diameter optical fiber core, the encoders showed a resolution of 12 and 18 pulses per revolution (PPR), respectively.

## 1. Introduction

In 1988, Fan et al. [1] first used semiconductor fabrication technologies to produce electrostatic micromotors. In 1990, Mehregany et al. [2] processed electrostatic wobble micromotors (variable-capacitance side-drive motors) using silicon-based processes. In 1991, Guckel et al. [3] applied the X-ray lithography and electroforming processes to the fabrication of microreluctance motors. In 1993, Guckel et al. [4] manufactured a 0.285 mm diameter reluctance rotor and used photodiodes to detect its rotational speed. In 1993, Wagner et al. [5] applied microcoils on a silicon substrate to drive sliding, rolling, and rotating permanent-magnet micromotors. In 1995, Ota et al. [6] used microcoils and cores made by lithography and electroforming processes to fabricate 1.2 mm diameter microgenerators. In 2006, Kim et al. [7] developed a 2 mm diameter permanent-magnet brushless motor and integrated it into a gear system to improve the output torque. In 2008, Waldschik et al. [8] integrated microcoils made by lithography into a 1 mm diameter micromotor with a polymer permanent magnet. In 2011, Merzaghi et al. [9] used micro-electromechanical systems (MEMS) to develop a 4 mm diameter axial-flux-type permanent-magnet brushless motor. In 2014, Wang et al. [10] applied a 1 mm diameter permanent-magnet brushless motor for intra-vessel ultrasound sonography. In 2014, Buttgenbach [11] reviewed different designs, fabrication processes, and applications of electromagnetic micromotors used in the past.

In 1991, Sawada et al. [12,13] developed an optical micro-encoder with laser diodes, photo-diodes, and microlenses and the scale had a grating pitch of 1.6 µm and a resolution of 0.01 µm. In 1997, Miyajima et al. [14] integrated a twin-beam vertical-cavity surface-emitting laser (VCSEL), photo-diodes, and micro lenses into an optical micro-encoder that had dimensions of 1.5 mm × 2.0 mm × 0.6 mm, a scale pitch of 20 µm, and a resolution of 0.1 µm. In 2003, Sawada et al. [15] worked out a hybrid microlaser encoder with a longer lifetime and smaller dimensions while retaining the same grating pitch of 1.6 µm. In 2006, Nagao et al. [16] manufactured a fiber-optic micro-encoder for a rotary-type electrostatic comb drive actuator; the encoder had a pitch of 20 µm and a resolution of 1 degree. In 2014, Lee et al. [17] integrated a reflective optical encoder into a smart microframe and the scale had a grating pitch of 10 µm and a resolution of 2.5 µm.

The review [11] showed that the number of permanent-magnet brushless micromotors that had been previously developed was very limited. The main reason for this is the difficulty associated with micromotor fabrication. Moreover, most motors have no sensor installed for feedback control, which deprived the motors of any precise positioning capabilities. The use of semiconductor technologies with modern optical micro-encoders has greatly reduced their sizes, but these encoders still cannot be installed in micromotors with diameters of less than 2 mm. This study proposed a rapid prototyping fabrication method that can make micromotors of different sizes. The process involves the use of a code disk and an optical fiber that can be placed inside 1 mm diameter micromotors, and provides the function of rotational angle feedback from an optical micro-encoder.

## 2. Micromotor Design and Analysis

### 2.1. Micromotor Designs

**Figure 1:**
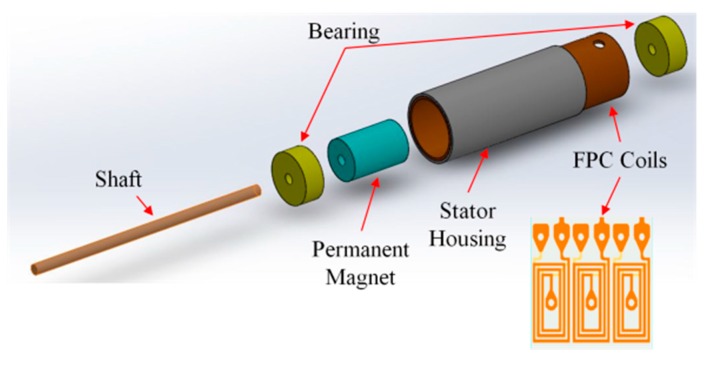
Exploded view of a micromotor, FPC: flexible print circuit.

A rapid prototyping fabrication method for integrating stator housing and flexible print circuit (FPC) coils was used to develop 1 mm and 1.5 mm diameter permanent-magnet synchronous micromotors (PMSM) in this study. The motors were two-pole, three-phase PMSM designs. Two different stator housing materials, copper and nickel cobalt, were applied. The advantage of using a non-magnetic copper shell is that the copper can be electroformed easily. The disadvantages of using a copper shell are that the micromotor has no holding torque and generates less torque. NdFeB magnets with a diameter of 0.64 mm and a length of 1 mm were used for 1 mm diameter motors. Magnets with a diameter of 1 mm and a length of 1.5 mm were used for 1.5 mm diameter motors. The three-phase concentrated-coil windings were designed with six turns and 10 turns for 1 mm and 1.5 mm motors, respectively. The air gaps were 0.1 mm and 0.2 mm, respectively. The specifications of 1 mm and 1.5 mm diameter motors are shown in Table 1. An exploded view of the motors is given in Figure 1.

### 2.2. Electromagnetic Analysis of Micromotors

**Figure 2:**
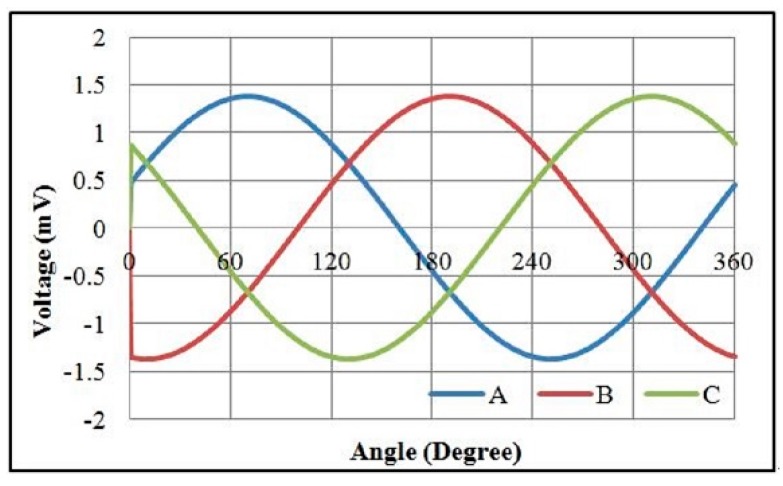
Back electromotive force (BEMF) of a 1 mm diameter motor with a Cu shell at 20,000 RPM.

**Figure 3:**
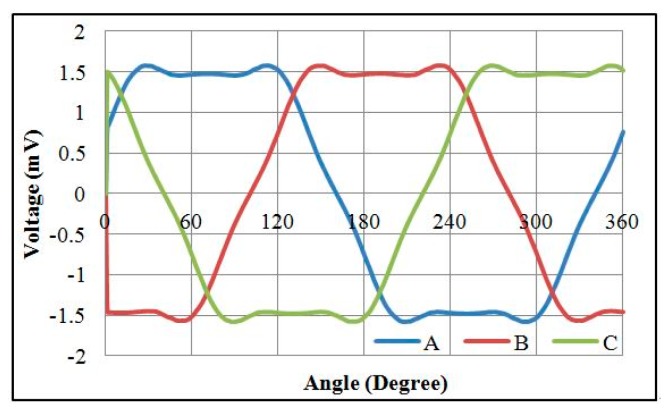
BEMF of a 1 mm diameter motor with a NiCo shell at 20,000 RPM.

**Figure 4:**
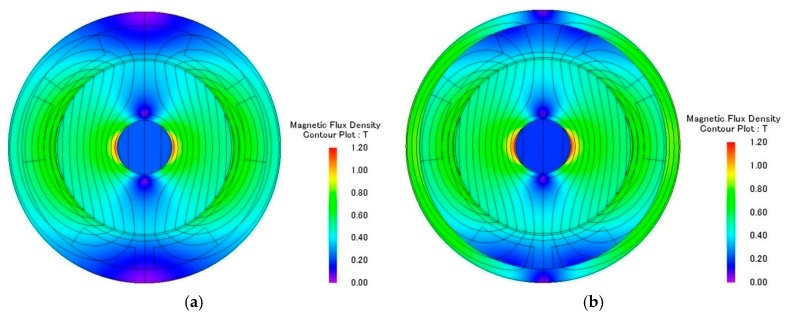
(**a**) Magnetic flux density of a 1 mm diameter motor with a Cu shell; (**b**) Magnetic flux density of a 1 mm diameter motor with a NiCo shell.

**Figure 5:**
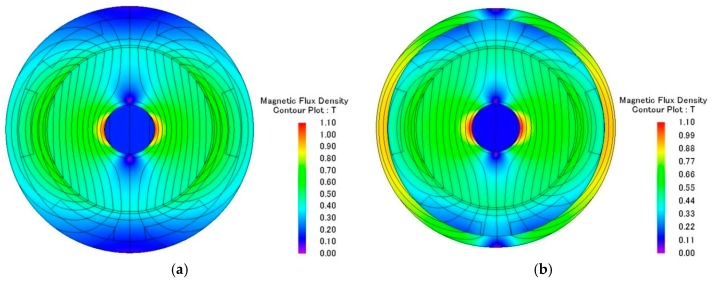
(**a**) Magnetic flux density of a 1.5 mm diameter motor with a Cu shell; (**b**) Magnetic flux density of a 1.5 mm diameter motor with a NiCo shell.

The electromagnetic analysis of the motors was conducted using JMAG software developed by the JSOL Corporation (Tokyo, Japan). Both 1 mm and 1.5 mm diameter motors were analyzed using 2D and 3D finite element models. The differences in output torque and back electromotive force (BEMF) between 2D and 3D models were less than 6%, so the 2D model was used for this study. The motors were driven by single-phase excitation. The output torque was calculated at the input current of 0.1 A to 0.3 A. The BEMF was evaluated at a rotational speed of 5000 RPM to 20,000 RPM. Two types of stator housing materials, copper and nickel cobalt, were examined and compared. The nickel cobalt shell was made by electroforming and had a saturation flux density of 0.5 T and a relative permeability of 600. For 1 mm diameter motors, the maximum measurements of output torque were 153 nN∙m and 213 nN∙m for copper and nickel cobalt shells, respectively. The average flux densities in the air gap were 0.5 T and 0.6 T, respectively. The BEMF measurements at 20,000 RPM were 1.08 mV and 1.51 mV for copper and nickel cobalt shells, respectively, and are shown in Figure 2 and Figure 3. The analytical results for 1 mm and 1.5 mm diameter motors are shown in Table 2. The magnetic flux densities of 1 mm diameter motors with either copper or nickel cobalt shells at maximum torque are shown in Figure 4. The results of 1.5 mm diameter motors are shown in Figure 5.

## 3. Development of an Optical Rotary Encoder

This study developed a reflective optical rotary encoder for a micromotor. Since typical optical detectors cannot fit into micromotors, optical fiber was used to transmit optical detection signals. The reflective code disk was assembled on a rotating shaft for angle measurement. The optical encoder consists of five components; the laser light source, an optical detector, a 1 × 2 coupler/splitter, optical fiber, and the code disk. The 1 × 2 coupler/splitter splits the reflective optical signals from the code disk into the optical detector. Bidirectional optical signal transmission was conducted via optical fiber, which can be embedded in the limited space of the micromotor. The laser light source and optical detector can be placed a long distance away without being restricted by the micromotor.

**Figure 6:**
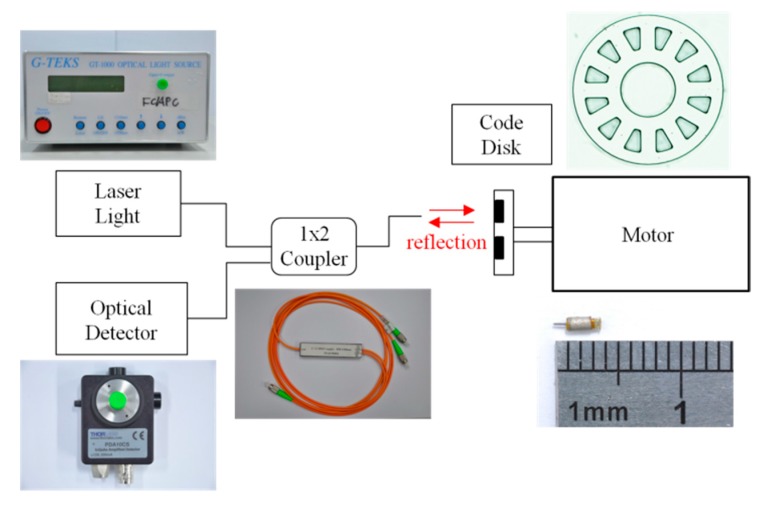
Micro-optical encoder system framework.

The code disk was designed for a 50 µm diameter optical fiber core. The code disk of a 1 mm motor has an outer diameter of 0.64 mm with 12 reflection gratings. The code disk of a 1.5 mm motor has an outer diameter of 1 mm with 18 reflection gratings. The optical rotary encoder system framework is shown in Figure 6. The system can be used to detect rotational angle and speed.

**Figure 7:**
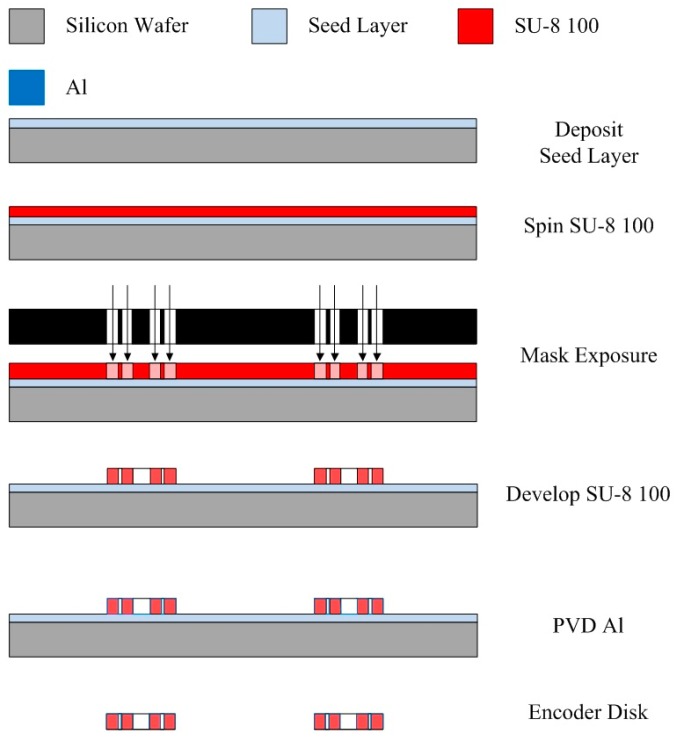
Micro-optical encoder system framework.

**Figure 8:**
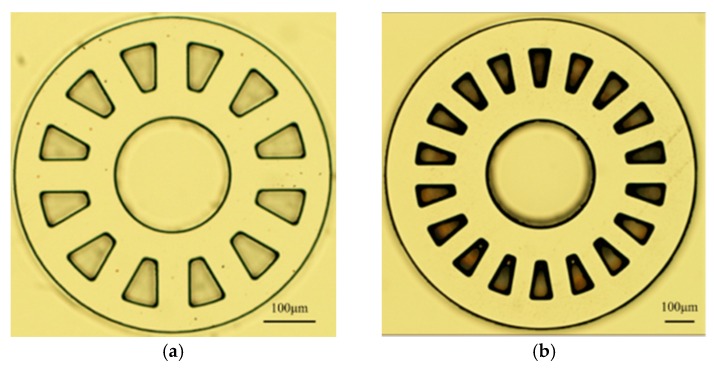
(**a**) Code disk with 12 gratings for 1 mm diameter motors; (**b**) Code disk with 18 gratings for 1.5 mm diameter motors.

The code disk was made of SU-8 100 photo-resist and aluminum thin-film. The fabrication process is as follows: First, a silicon wafer was sputtered with a platinum seed layer and then spun with a SU-8 100 photo-resist coating. The SU-8 100 was soft-baked and exposed with the code disk images using a photo mask. After hard-baking, the SU-8 was developed and left with the code disk structures. The aluminum deposited on the code disk structures served as an optical reflector. Finally, the code disks were removed from the seed layer. The fabrication procedure for the code disk is shown in Figure 7. The code disks of 1 mm and 1.5 mm motors are illustrated in Figure 8.

## 4. Micromotor Fabrication and Testing

### 4.1. Micromotor Fabrication

**Figure 9:**
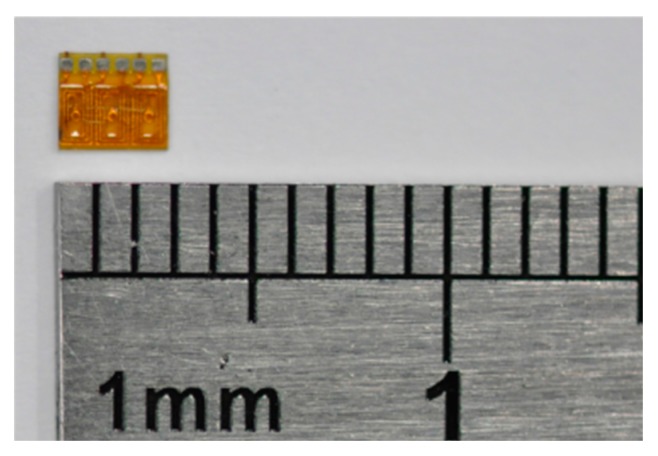
FPC board of a 1 mm diameter motor.

This study proposed an integrated electroforming method for combining FPC coils and stator housing to simplify the fabrication of micromotors. The coils were made using flexible double sided FPC consisting of a polyimide (PI) base material and two layers of copper foil. The FPC coils for a 1 mm diameter motor, as shown in Figure 9, had lengths of 2.56 mm, widths of 2.86 mm, and thicknesses of 80 µm. Each coil had a total of six turns with a copper foil thickness of 20 µm, a line width of 50 µm, and a line space of 50 µm.

**Figure 10:**
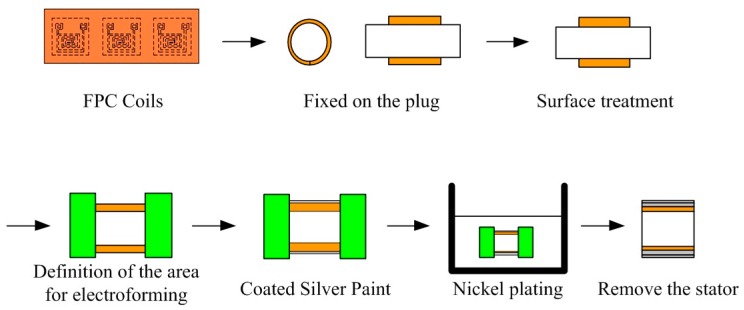
Integrated electroforming processes of FPC coils and stator housing.

Taking advantage of the highly flexible FPC, the stator housing structure can be electroformed on the outside of circularly wound FPC board. The fabrication procedure for integrating flexible FPC coils and stator housing is as follows: First, the flexible FPC board was bent into a cylindrical shape and fixed using adhesive tape on a precision plug gauge in order to guarantee the roundness of the stator’s inner diameter and to avoid an expansion of the air gap which would cause a reduction in the motor’s output torque. Prior to electroforming, the FPC surface was subjected to an alkaline cleaning, neutralization, activation, and drying process. A conductive paint was applied to the selective surface area for electroforming. The stator shell was electroformed with copper or a nickel cobalt alloy. Finally, the flexible FPC coils and the stator housing were removed, which completed the integrated electroforming procedure. The whole processes are shown in Figure 10.

The NdFeB permanent magnets used for 1 mm and 1.5 mm micromotors were made of N48H and 38UH materials from the Teslar Technology Co., Ltd. (Taichung, Taiwan). Both magnets were experimentally tested to ensure their magnetic properties. The N48H had a higher residual magnetism Br of 1.4 T, a coercivity Hcb of 1060 kA/m and a magnetic energy product (BH) maximum of 370 kJ/m^3^. The 38UH material had a Br of 1.2 T, an Hcb of 960 kA/m and a magnetic energy product (BH) maximum of 290 kJ/m^3^. A 1 mm motor using a stronger permanent magnet can generate more torque with a higher flux density in the air gap.

The bearings were made of brass for both motors because there is no ball bearing available and ruby bearings are too expensive. The 1.5 mm motors used both single-bearing and two-bearing designs but 1 mm motors used only two-bearing design. The performance of different bearing designs was evaluated using a run-out test. The motor shafts were made of a stainless steel material.

It is easy to manufacture a micromotor with a non-magnetic copper shell because the copper can be electroformed using a simple experimental setup. However, the micromotor with the copper shell had no holding torque and generated less output torque. An advantage of using of a magnetic nickel cobalt shell is that the nickel cobalt alloy has a higher magnetic saturation value than nickel. The cobalt content can increase the hardness and the strength, so the plating conditions such as solution composition, current density, and temperature are critical to avoiding large internal stress. Using this technology, micromotors with various sizes and output characteristics can be made and applied in different scenarios.

### 4.2. Micromotor Assembly

**Figure 11:**
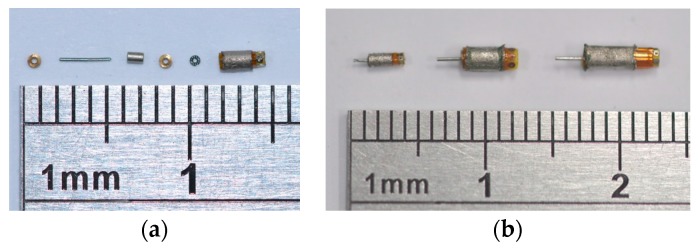
(**a**) 1 mm diameter motor components; (**b**) Assembled motors with 1 mm and 1.5 mm diameters.

The micromotors were manually assembled under a digital microscope. Tweezers and two precision plug gauges were used to assist the processes. First, the permanent magnet and the shaft were connected with a press fit. Second, one bearing was placed in the stator housing with the assistance of gauges with the same diameter as was used in the electroforming process. The other bearing was put through the shaft next to the permanent magnet. The shaft was guided by the bearing already in place and all the components were pushed by the gauge into the stator housing. The code disk was fastened to the shaft with adhesive. Finally, the optical fiber base was stuck to the optical fiber and attached to the stator housing. Components of the permanent-magnet synchronous motor with an optical rotary encoder are shown in Figure 11a. Assembled micromotors with 1 mm and 1.5 mm diameters are shown in Figure 11b.

## 5. Micromotor and Optical Encoding Board Testing

**Figure 12:**
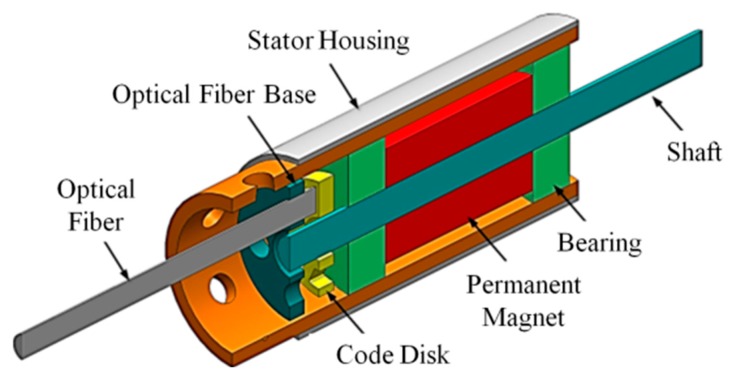
Assembly drawing of a micromotor with optical encoder.

**Figure 13:**
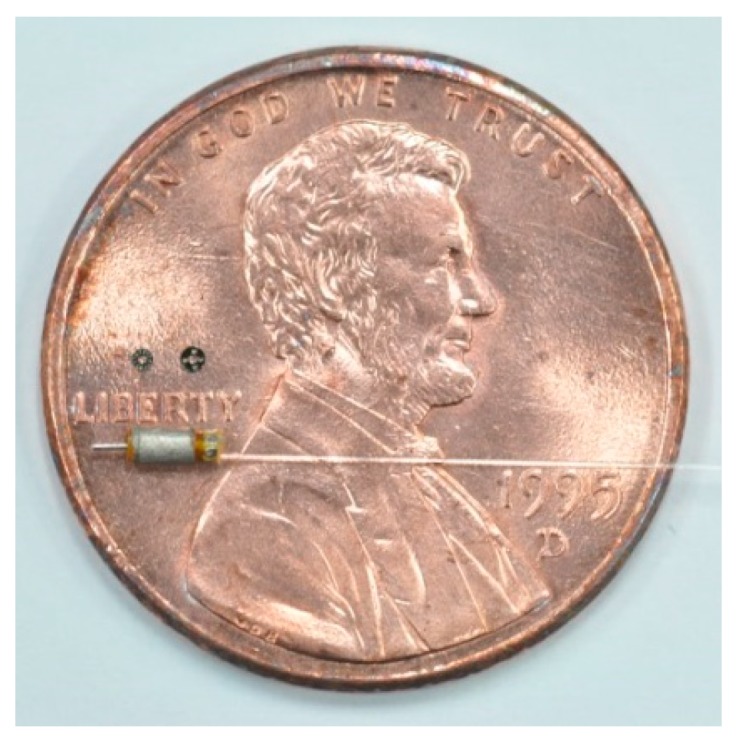
A 1 mm diameter micromotor with an optical encoder.

A double-bearing motor design was used for the 1 mm diameter micromotor developed in this study. A model of a double-bearing motor is shown in Figure 12. A single-bearing motor design was used for the 1.5 mm diameter micromotor because simplified components could allow for ease of assembly. A stator-housing-integrating flexible coil was used to reduce the number of manufacturing procedures. This improved the assembly precision and reduced the difficulties associated with the installation of an encoder in a micromotor, allowing application of the micromotor in precision positioning control and reducing development costs. The motor with an encoder is illustrated in Figure 13. The optical rotary micro-encoder was tested before connection to the permanent-magnet synchronous micromotor. Experiments were conducted to find BEMF, output torque, and run-out.

### 5.1. Measurement of Motor Characteristics

#### 5.1.1. Characteristics of 1 mm Diameter Motors

**Figure 14:**
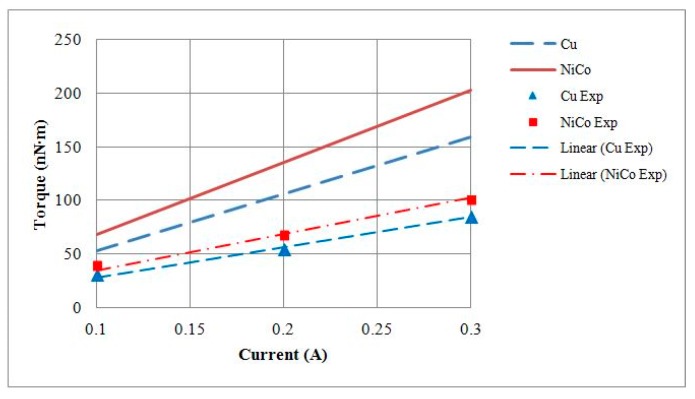
Theoretical and experimental output torque of 1 mm diameter motors with Cu or NiCo shells.

Output torque testing of the motor was conducted by hanging weights at its shaft. At a current of 0.3 A, maximum weights of 0.0614 g and 0.0730 g were measured in 1 mm motors with either a copper shell or a nickel cobalt shell, respectively. Our calculations showed that the maximum torque measurements were 84.97 nN∙m and 101.10 nN∙m, respectively, and the torque constant (*K*_T_) values were equal to 282.52 nN∙m/A and 343.27 nN∙m/A, respectively. A comparison of the *K*_T_ values is provided in Table 3. The torque–current curves plotted with the theoretical and experimental values are given in Figure 14.

**Figure 15:**
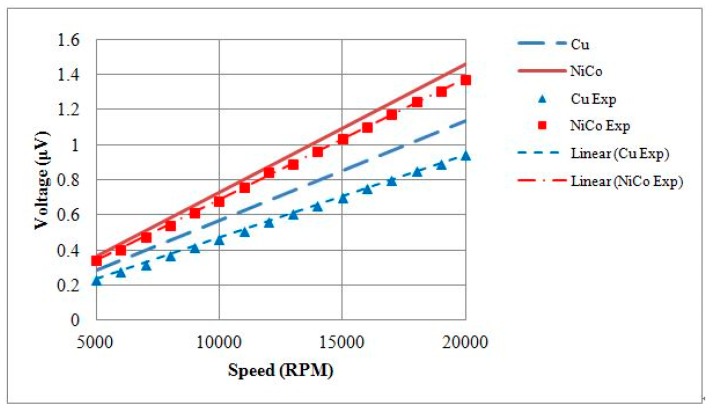
Theoretical and experimental BEMF values of 1 mm diameter motors with Cu or NiCo shells.

For motor BEMF testing, voltages were measured at various speeds. At a rotational speed of 20,000 RPM, the BEMF root-mean-square (RMS) voltages generated by 1 mm motors with a copper shell or a nickel cobalt shell were 0.9452 mV and 1.3708 mV, respectively. The BEMF constant (*K*_V_) values of the motors were 448.30 nV/(rad/s) and 656.30 nV/(rad/s), respectively. A comparison of the *K*_V_ values is provided in Table 3. The rotational speed–BEMF curves based on theoretical and experimental values are shown in Figure 15.

#### 5.1.2. Characteristics of 1.5 mm Diameter Motors

**Figure 16:**
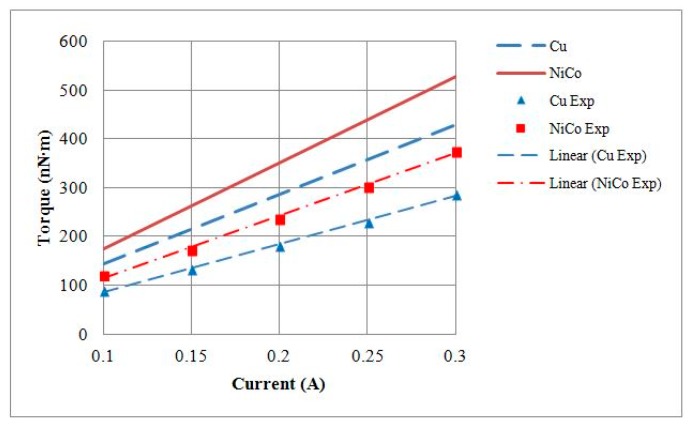
Theoretical and experimental output torque values of 1.5 mm diameter motors with Cu or NiCo shells.

**Figure 17:**
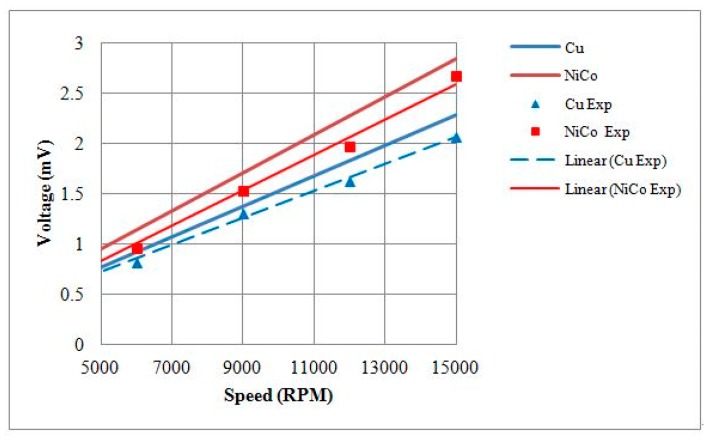
Theoretical and experimental BEMF values of 1.5 mm diameter motors with Cu or NiCo shells.

The maximum torque measurements of 1.5 mm motors with either a copper shell or a nickel cobalt shell were 288 nN∙m and 376 nN∙m, with *K*_T_ values of 929.33 nN∙m/A and 1217.55 nN∙m/A, respectively. A comparison of the *K*_T_ values is provided in Table 4. At a rotational speed of 15,000 RPM, the BEMF RMS voltages of 1.5 mm motors with either copper or nickel cobalt shells were 2.067 mV and 2.674 mV, respectively. The *K*_V_ values of the motors were 1323.5 nV/(rad/s) and 1638.4 nV/(rad/s), respectively. A comparison of *K*_V_ values is provided in Table 4. Finally, the output torque and the BEMF testing results were compared against theoretical models, and the current–torque curves are shown in Figure 16 and rotational speed–BEMF curves are shown in Figure 17.

### 5.2. Motor Run-Out Test

A Keyence laser displacement sensor (Keyence LK-G10, Keyence, Osaka, Japan) was used in the motor run-out test. A laser light was projected on the motor shaft and part of the light was reflected back to the laser displacement sensor. When the motor shaft was rotated off-center, the run-out influenced the laser light output signals. A 1.5 mm motor with a nickel cobalt shell was tested with three different bearing setups: a single-bearing design with two different thicknesses, 0.5 mm and 1 mm, and a double-bearing design with thicknesses of 0.5 mm each. The results showed that the run-out was smaller in the single-bearing design with the larger thickness of 1 mm. The results of the run-out test for the 1.5 mm motor are provided in Table 5.

The run-out was tested in 1 mm motors with either a copper shell or a nickel cobalt shell at a rotational speed of 20,000 RPM. In 1 mm motors, a double-bearing design was applied. The run-out test results for 1 mm motors are shown in Table 6.

### 5.3. Testing Motors with a Rotary Encoder

**Figure 18:**
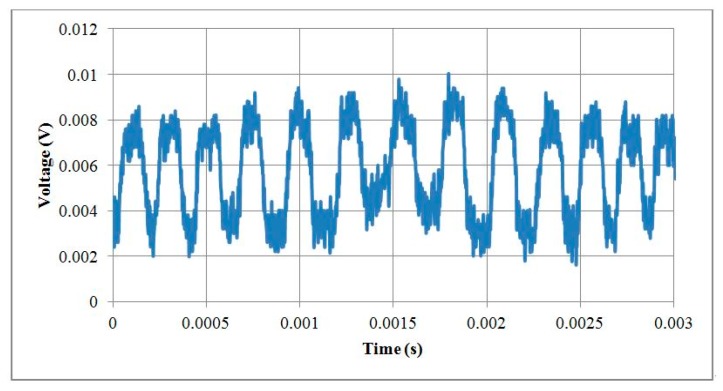
Output voltage signals of an optical encoder within a 1 mm motor.

**Figure 19:**
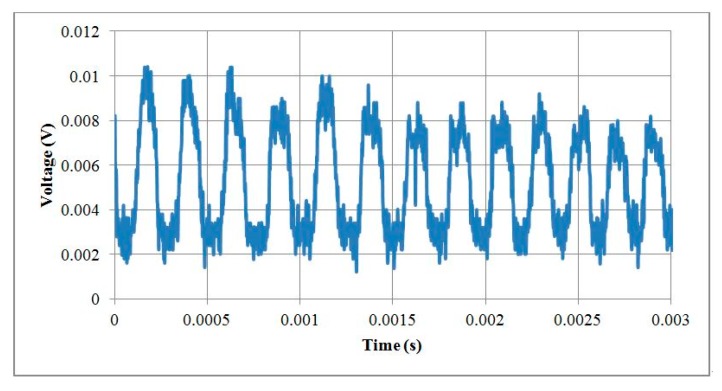
Output voltage signals of an optical encoder within a 1.5 mm motor.

The 1 mm and 1.5 mm micromotors were tested with their optical rotary encoders. The pulses per revolution for the encoders were 12 PPR and 18 PPR for 1 mm and 1.5 mm motors, respectively. The 1 mm motor was tested at a rotational speed of 20,000 RPM and the output voltage signals of the encoder are illustrated in Figure 18. The 1.5 mm motor was tested at a rotational speed of 15,000 RPM and the output voltage signals of the encoder are illustrated in Figure 19. The encoder resolution was limited by the number of reflection gratings in the code disks because the code disks were designed based on a 50 µm diameter optical fiber core.

## 6. Discussion

1. Due to the difficulties in measuring magnetic flux density in micromotors, only the motor constant values could be tested to support the theoretical analysis. A comparison of the theoretical and the experimental *K*_V_ and *K*_T_ data showed that losses were caused by friction, which led to lower efficiency.
2. Brass was used to make the micromotor bearings developed in this study. The run-out was relatively large. The use of bearings made from ruby or other high-quality materials could solve the issue of the high run-out in the motors.
3. Due to the restrictions on the FPC, such as line width, line space, and thickness, the maximum current was only 0.3 A. This resulted in a lower maximum flux density, leading to a limited performance in the output torque.
4. The run-out in the developed micromotors was relatively large. Moreover, the core of the optical fiber was 50 µm, which limited the number of reflection gratings of the encoder. This led to poor signal transmission and sensor resolution. Further improvements are required with respect to optical fiber equipment and the run-out in order to increase the resolution.

## 7. Conclusions

This study proposed an innovative method to ease the production of micromotors in highly customized or low-volume conditions. The stator housing was made on the external surface of FPC coils using an electroforming process. This facilitated the manufacturing of motors of different sizes and removed the necessity to assemble stator housings. Moreover, this study developed a reflective rotary micro-encoder using photolithography and sputtering, which enabled the application of the micromotors in precision positioning. The 1 mm and 1.5 mm diameter permanent-magnet synchronous motors with optical encoders were developed successfully. At a maximum current of 0.3 A, the 1 mm motors produced torque of 71.66 nN∙m and reached a no-load rotational speed of 24,000 RPM. The 1 mm motor was equipped with a 0.64 mm rotary micro-encoder to measure its rotational angle and speed. With this rapid prototyping fabrication method, the authors have completed the development of a series of motors with diameters from 1 mm to 7 mm.

## Figures and Tables

**Table 1:** Specifications of permanent magnet (PM) synchronous micromotors.

**Mechanical Specifications of a 1 mm Diameter Motor**					
Stator	External Diameter	1 mm	Rotor	External Diameter	0.64 mm
	Internal Diameter	0.74 mm		Internal Diameter	0.2 mm
	Length	2.5 mm		Length	1 mm
	Air gap	0.1 mm		Number of Poles	2
**Mechanical Specifications of a 1.5 mm Diameter Motor**					
Stator	External Diameter	1.5 mm	Rotor	External Diameter	1 mm
	Internal Diameter	1.4 mm		Internal Diameter	0.3 mm
	Length	4.1 mm		Length	1.5 mm
	Air Gap	0.2 mm		Number of Poles	2
**Electrical Specifications of Motors**					
Number of Phases		3	Exciting Current		0.3 A
Line Width 1 mm/1.5 mm		50 µm/60 µm	Line Space 1 mm/1.5 mm		50 µm/60 µm
Coil Turns 1 mm/1.5 mm		6 turns/10 turns	Coil Resistance 1 mm/1.5 mm		5.225 Ω/1 Ω

**Table 2:** Characteristics of 1 mm and 1.5 mm motors with Cu or NiCo shells based on JMAG analysis.

Diameter	Shell	Torque (nN∙m)	BEMF (mV)	Power (µW)
1 mm	Cu	159	1.14	89
	NiCo	203	1.46	108
1.5 mm	Cu	430	3.04	154
	NiCo	527	3.78	206

**Table 3:** Specifications of 1 mm diameter PM synchronous micromotors.

Motor Type & Model	*K*_T_ (nN∙m/A)	*K*_V_ (nV/(rad/s))
Copper shell analysis	530.5	544.2
Copper shell test	282.5	448.3
Nickel-cobalt shell analysis	677.0	695.5
Nickel-cobalt shell test	343.3	656.3

**Table 4:** Specifications of 1.5 mm diameter PM synchronous micromotors.

Motor Type & Model	*K*_T_ (nN∙m/A)	*K*_V_ (nV/(rad/s))
Copper shell analysis	1432.3	1452.2
Copper shell test	929.3	1323.5
Nickel-cobalt shell analysis	1755.3	1806.7
Nickel-cobalt shell test	1217.6	1638.4

**Table 5:** Run-out of 1.5 mm diameter PM synchronous micromotors.

Bearing Type	Run-Out
Double-bearing	5 µm
Single-bearing (*L* = 0.5 mm)	20 µm
Single-bearing (*L* = 1 mm)	2 µm

**Table 6:** Run-out of 1 mm diameter PM synchronous micromotors.

Housing Material	Run-Out
Copper	20 µm
Nickel-cobalt	40 µm
